# Home-Based Versus Mobile Clinic HIV Testing and Counseling in Rural Lesotho: A Cluster-Randomized Trial

**DOI:** 10.1371/journal.pmed.1001768

**Published:** 2014-12-16

**Authors:** Niklaus Daniel Labhardt, Masetsibi Motlomelo, Bernard Cerutti, Karolin Pfeiffer, Mashaete Kamele, Michael A. Hobbins, Jochen Ehmer

**Affiliations:** 1 Clinical Research Unit, Medical Services and Diagnostic, Swiss Tropical and Public Health Institute, University of Basel, Basel, Switzerland; 2 SolidarMed Lesotho, Seboche Hospital, Butha-Buthe, Lesotho; 3 Faculty of Medicine, University of Geneva, Geneva, Switzerland; 4 SolidarMed Switzerland, Lucerne, Switzerland; 5 SolidarMed Lesotho, Paray Hospital, Thaba-Tseka, Lesotho; McGill University Health Centre, Canada

## Abstract

**Background:**

The success of HIV programs relies on widely accessible HIV testing and counseling (HTC) services at health facilities as well as in the community. Home-based HTC (HB-HTC) is a popular community-based approach to reach persons who do not test at health facilities. Data comparing HB-HTC to other community-based HTC approaches are very limited. This trial compares HB-HTC to mobile clinic HTC (MC-HTC).

**Methods and Findings:**

The trial was powered to test the hypothesis of higher HTC uptake in HB-HTC campaigns than in MC-HTC campaigns. Twelve clusters were randomly allocated to HB-HTC or MC-HTC. The six clusters in the HB-HTC group received 30 1-d multi-disease campaigns (five villages per cluster) that delivered services by going door-to-door, whereas the six clusters in MC-HTC group received campaigns involving community gatherings in the 30 villages with subsequent service provision in mobile clinics. Time allocation and human resources were standardized and equal in both groups. All individuals accessing the campaigns with unknown HIV status or whose last HIV test was >12 wk ago and was negative were eligible. All outcomes were assessed at the individual level. Statistical analysis used multivariable logistic regression. Odds ratios and *p*-values were adjusted for gender, age, and cluster effect.

Out of 3,197 participants from the 12 clusters, 2,563 (80.2%) were eligible (HB-HTC: 1,171; MC-HTC: 1,392). The results for the primary outcomes were as follows. Overall HTC uptake was higher in the HB-HTC group than in the MC-HTC group (92.5% versus 86.7%; adjusted odds ratio [aOR]: 2.06; 95% CI: 1.18–3.60; *p* = 0. 011). Among adolescents and adults ≥12 y, HTC uptake did not differ significantly between the two groups; however, in children <12 y, HTC uptake was higher in the HB-HTC arm (87.5% versus 58.7%; aOR: 4.91; 95% CI: 2.41–10.0; *p*<0.001). Out of those who took up HTC, 114 (4.9%) tested HIV-positive, 39 (3.6%) in the HB-HTC arm and 75 (6.2%) in the MC-HTC arm (aOR: 0.64; 95% CI: 0.48–0.86; *p* = 0.002). Ten (25.6%) and 19 (25.3%) individuals in the HB-HTC and in the MC-HTC arms, respectively, linked to HIV care within 1 mo after testing positive. Findings for secondary outcomes were as follows: HB-HTC reached more first-time testers, particularly among adolescents and young adults, and had a higher proportion of men among participants. However, after adjusting for clustering, the difference in male participation was not significant anymore.

Age distribution among participants and immunological and clinical stages among persons newly diagnosed HIV-positive did not differ significantly between the two groups. Major study limitations included the campaigns' restriction to weekdays and a relatively low HIV prevalence among participants, the latter indicating that both arms may have reached an underexposed population.

**Conclusions:**

This study demonstrates that both HB-HTC and MC-HTC can achieve high uptake of HTC. The choice between these two community-based strategies will depend on the objective of the activity: HB-HTC was better in reaching children, individuals who had never tested before, and men, while MC-HTC detected more new HIV infections. The low rate of linkage to care after a positive HIV test warrants future consideration of combining community-based HTC approaches with strategies to improve linkage to care for persons who test HIV-positive.

**Trial registration:**

ClinicalTrials.gov NCT01459120

*Please see later in the article for the Editors' Summary*

## Abstract 2

Niklaus Labhardt and colleagues investigate how different HIV testing and counseling strategies, based on home visits or mobile clinics, reach different populations in a rural African setting.

*Please see later in the article for the Editors' Summary*

## Editors' Summary

**Background:**

Annually, about 2.3 million people become newly infected with HIV, the virus that causes AIDS by gradually destroying CD4 cells and other immune system cells, thereby leaving HIV-infected individuals susceptible to other serious infections. HIV can be transmitted through unprotected sex with an infected partner, from an HIV-positive mother to her unborn child, or through the injection of drugs with shared needles. Infection with HIV is usually diagnosed by looking for antibodies to HIV in the blood or saliva. After diagnosis, the progression of HIV infection is monitored by regularly counting the number of CD4 cells in the blood. Initiation of antiretroviral therapy (ART)—a combination of drugs that keeps HIV replication in check but that does not cure the infection—is recommended when an individual's CD4 count falls below 500 cells/µl or when he or she develops signs of advanced or severe disease, such as unusual infections.

**Why Was This Study Done?:**

To control HIV/AIDS, HIV transmission needs to be reduced, and ART delivery needs to be increased. In settings of high HIV prevalence, universal coverage of HIV testing and counseling (HTC) is essential if these goals are to be met. Unfortunately, many people refuse “facility-based” HTC (HTC delivered at health care facilities) because they fear stigmatization and discrimination. Moreover, many people in resource-limited settings rarely visit health care facilities. Community-based HTC may be one way to increase the uptake of HTC, particularly among populations that are hard to reach, such as men and first-time testers, but which form of community-based HTC will be most effective? In this cluster-randomized trial, the researchers ask whether home-based HTC (HB-HTC)—community-based HTC in which health care workers go door-to-door to offer HTC to people in their own home—results in a higher uptake of HTC than HTC delivered through community gatherings and mobile clinics (MC-HTC) in two rural areas in Lesotho. Nearly a quarter of adults are HIV-positive in Lesotho, but only 61% of people who need ART currently receive treatment. A cluster-randomized trial compares outcomes in groups (clusters) of people chosen at random to receive different interventions.

**What Did the Researchers Do and Find?:**

The researchers allocated 12 clusters, each comprising a health center and its catchment area, to the HB-HTC or MC-HTC intervention. In the HB-HTC arm (1,171 participants), HTC teams going door-to-door delivered a multi-disease campaign that included HTC to five villages in each cluster. In the MC-HTC arm (1,392 participants), the multi-disease campaign was delivered at community gatherings with subsequent service provision in mobile clinics. Overall, HTC uptake was higher in the HB-HTC arm than in the MC-HTC arm (92.5% and 86.7% uptake, respectively). Among participants aged ≥12 years, there was no significant difference in HTC uptake between the arms, whereas among children aged <12 years, HTC uptake was significantly higher in the HB-HTC arm than in the MC-HTC arm (87.5% versus 58.7%; a significant difference is a difference unlikely to have happened by chance). Among individuals who took up HTC, 3.6% and 6.2% tested positive for HIV in the HB-HTC arm and MC-HTC arm, respectively. In both arms, only a quarter of individuals who tested positive accessed HIV care within a month of their positive test result. Finally, HB-HTC reached more first-time testers (particularly among adolescents) and tended to reach more men than MC-HTC.

**What Do These Findings Mean?:**

These findings suggest that, in rural Lesotho, both HB-HTC and MC-HTC delivered as part of a multi-disease campaign can achieve a high uptake of HTC. Various aspects of the trial design (for example, the small number of clusters) may limit the accuracy of the findings reported here. Notably, however, these findings suggest that the choice between HB-HTC and MC-HTC should be guided by the objective of the HTC intervention in specific settings. Where equity of access is of concern and where increased HTC coverage, particularly among groups in which HTC coverage is generally poor (including men, first-time testers, and children), is paramount, HB-HTC may be the preferred option. By contrast, the MC-HTC approach may be more appropriate in settings where the detection of new HIV infections is the major goal. Finally, and importantly, the findings of this trial highlight the need for further research into strategies designed to improve the linkage between HIV testing and enrollment into care.

**Additional Information:**

Please access these websites via the online version of this summary at http://dx.doi.org/10.1371/journal.pmed.1001768.

- The World Health Organization provides information on all aspects of HIV/AIDS, including information on HIV counseling and testing (in several languages)
- Information is available from the US National Institute of Allergy and Infectious Diseases on HIV infection and AIDS
- NAM/aidsmap provides basic information about HIV/AIDS and summaries of recent research findings on HIV care and treatment
- Information is available from Avert, an international AIDS charity, on many aspects of HIV/AIDS, including information on the global HIV/AIDS epidemic, on HIV testing, and on HIV/AIDS in Lesotho
- The UK National Health Service Choices website provides information (including personal stories) about HIV/AIDS
- The “UNAIDS Report on the Global AIDS Epidemic 2013” provides up-to-date information about the AIDS epidemic and efforts to halt it
- Stories about living with HIV/AIDS are available through Avert and through healthtalk.org
- More information about this trial is available

## Introduction

The success of national HIV programs relies on widely accessible HIV testing and counseling (HTC) services, which are the first step towards control of the global HIV/AIDS epidemic [1]–[3]. While HTC represents the entry point to antiretroviral therapy (ART) for those in need, it also acts as a preventive intervention for those who test negative and for partners of persons testing HIV-positive and subsequently starting ART [4]–[6]. However, universal coverage of HTC has not been reached yet. Uptake of HTC in a standard clinical setting is usually low [7], particularly among men [8],[9]. Further, HTC at clinics remains limited to persons who attend medical facilities. Several studies have concluded that HTC provision outside clinical settings (community-based HTC) is feasible and acceptable and results in a higher uptake of HTC, particularly among populations that are usually hard to reach, such as men or first-time testers [7],[10]–[13]. There are different kinds of community-based HTC, including community gatherings around mobile clinics (mobile clinic HTC [MC-HTC]) and home-based HTC (HB-HTC). During HB-HTC, a team—most often consisting of a counselor and a nurse—goes door-to-door, visiting each household in the community and offering HTC to the household members. HB-HTC has recently been advocated by the World Health Organization [14]. The approach showed high acceptance [15] and is convenient to the community [16]–[19]. These qualities result in high uptake within rural areas and provide access to hard to reach populations [7]–[13],[20]–[22]. Sabapathy and colleagues conclude from a systematic review and meta-analysis of 21 observational studies in five African countries that HB-HTC could substantially increase awareness of previously undiagnosed persons about their HIV status [15].

However, there are limited data comparing HB-HTC to facility-based HTC [22] and—to our knowledge—no randomized trials comparing HB-HTC to other community-based HTC delivery approaches such as MC-HTC. A Cochrane review in 2010 concluded that although HB-HTC may have the potential to scale up HTC in low-resource settings, not enough data were available to recommend its implementation on a large scale until studies comparing HB-HTC to more conventional approaches were conducted [23].

In Lesotho, where the adult HIV prevalence is 23.6% and the ART coverage rate is only 61% [2],[24], strategies to provide universal HTC are urgently needed. With the aim of testing which programmatic approach would be more effective for a rural, high-prevalence setting, this cluster-randomized trial compared two community-based HTC interventions: HB-HTC and MC-HTC.

## Methods

### Ethics Statement

The study protocol was approved by the National Research and Ethics Committee of the Ministry of Health and Social Welfare of Lesotho. All individuals gave oral and written consent prior to participating in the study. In the case of children <18 y of age, the caregiver gave oral and written consent.

### Study Hypothesis, Design, and Setting

The study's main hypothesis was that the HB-HTC approach would result in a higher uptake of HTC than the MC-HTC approach among individuals accessing the multi-disease campaigns.

The study was an open-label, two-armed cluster-randomized trial conducted in two rural catchment areas of Lesotho (Seboche Hospital and Paray Hospital) from 17 October to 25 November 2011. The catchment area of Seboche Hospital (district of Butha-Buthe) in northern Lesotho has an estimated population of 55,000 inhabitants served by five primary health care centers. The catchment area of the Paray Hospital is in the mountainous district of Thaba-Tseka in central Lesotho. It has a population of about 77,000 inhabitants served by seven primary health care centers. HTC services are available in all health centers of the study area. The population of both districts lives mainly from subsistence farming. According to the Lesotho Demographic Health Survey 2009, the adult HIV prevalence in Butha-Buthe and Thaba-Tseka is 15.9% and 20.1%, respectively [24]. The government of Lesotho estimated in 2009 that 25.7% of adult women who were HIV-positive and 41% of men who were HIV-positive had never been tested for HIV [25]. HTC coverage in the two districts is comparable, with about 60% of adult women and 40% of men having ever had an HIV test [24]. SolidarMed, a Swiss non-governmental organization, has been supporting the national HIV program, including HTC delivery, in the study area since 2005.

With the Know Your Status Campaign, Lesotho introduced a national concept to standardize HTC services in 2006 [26]. Although the campaign led to an increase in HTC coverage, there were concerns related to the quality of services, in particular with regard to obtainment of informed consent, ensuring confidentiality, and linkages to services and support [27]. Nevertheless, the campaign provided an important national framework for task shifting of HTC from health care professionals to community and lay counselors, thus allowing delivery of decentralized and community-based HTC. In Lesotho, community and lay counselors are non-medical personnel who have undergone a 6-wk standardized training in HTC provided by the Ministry of Health and Social Welfare (or, since 2012, the Ministry of Health) of Lesotho. Professional and senior counselors supervise the lay counselors at the facility and district levels, respectively. In Lesotho, mainly two community-based HTC approaches are implemented: HB-HTC and MC-HTC at community gatherings, locally called “pitso.” The latter has become very common and is also used to provide preventive medical services such as immunization, antenatal care consultations, and malnutrition screening among children.

### Allocation and Randomization

Since both HB-HTC and MC-HTC have an impact on the interaction between individuals of the same community, allocation and randomization were based on clusters. One health center with its catchment area formed a cluster. Paray Hospital has seven health centers affiliated to it, and Seboche Hospital has five. All 12 centers were included in the study, to form 12 clusters. The clusters were stratified and paired using data from routine HTC activities at their clinics and subsequently randomized into the two study groups. Pairing before randomization was based on the following three indicators from routine monitoring data: monthly routine facility-based HTC, monthly positive HTC results, and monthly enrollment in care of persons newly diagnosed HIV-positive. In addition, the affiliated hospital (Seboche or Paray) was taken into account during the pairing of clusters. After the pairing of clusters, pairs were randomized into the two study groups. In one group, HTC was provided within multi-disease campaigns at community gatherings around a mobile clinic (MC-HTC group). In the other group, HTC was provided within the same multi-disease campaigns, but using a door-to-door approach (HB-HTC group).

During the official kick-off meeting with the study teams and representatives of both districts, an independent person randomly allocated clusters by means of throwing a coin. As a result, six clusters (one of each pair) were assigned to the HB-HTC group, while the other six were assigned to the MC-HTC group. Before random allocation of the clusters to the interventions, the team of each health center provided a list with ten villages within its catchment area eligible for an HTC campaign according to the following criteria: villages with at least 25 households that were geographically clearly confined to the catchment area and located at a distance of three to 20 km from the facility. Five of the ten eligible villages in each cluster were randomly selected to receive the intervention; the other five served as control villages, with no HTC campaign held during the study period. The numbers of individuals newly tested HIV-positive residing in the control villages of both groups and identified through routine facility-based HTC in both study groups were collected as part of the descriptive cluster analysis during a period of 28 d after the campaigns held in the intervention villages.

Within each cluster, the health center conducted a 1-d HTC campaign (MC-HTC or HB-HTC) in each of the five selected villages, resulting in five 1-d campaigns per cluster and 30 campaigns per study arm. Within each cluster pair, the five HTC campaigns per cluster were held simultaneously. The duration of the intervention phase was therefore 6 wk, and the total number of HTC campaigns performed was 60.

### Services Provided during the Campaigns

Irrespective of the study arm, services provided during all HTC campaigns followed the principle of a multi-disease campaign and included the following: clinical tuberculosis screening and measurement of weight and height for all individuals, family planning, blood pressure and blood sugar measurement for adults, and provision of vitamin A, standard immunizations, and deworming for eligible children and women. In line with general practice in the country, HTC was performed by trained lay counselors under supervision of the hospital's professional counselor as well as the district's senior counselor. HTC followed the national guidelines [27]. Testing was done with Alere Determine. In the case of a positive result, the test was confirmed by Alere DoubleCheck Gold. In case of incongruence between Alere Determine and Alere DoubleCheck Gold, SD Bioline HIV was used as a third test. In the case of a confirmed positive HIV test result, CD4 count was determined on site, using a point-of-care CD4 test (Alere Pima). Clinical WHO staging was done by the nurse on site. During the whole process of pre-test counseling, testing, and post-test counseling, the participant and counselor stayed together in the same room in order to ensure that all participants who took up HTC received full HTC including post-test counseling. In compliance with the national guidelines, the name, age, and village of participants who took up HTC were registered in the national HTC registers. In addition, participants received a study number in order to allow anonymous processing of data. After post-test counseling, persons newly detected as HIV-positive were referred to their nearest health center using the national standard referral form. If they agreed, their names, addresses, and cell-phone numbers—if available—were noted and sent to the health center to allow tracing in case they did not enroll for care within 1 mo after their test.

### Human Resources and Time Allocation during the Campaigns

Human resources and time allocation were equal in both study arms. Each campaign was conducted by a team of five persons: one professional counselor, two nurses, and two lay counselors. This team divided into two sub-teams of one lay counselor and one nurse who provided the services together. The two sub-teams were supervised by the professional counselor. The campaigns started at 9:00 a.m. and lasted until 5:00 p.m. The time slot was limited because of feasibility issues. First, travel time to some of the villages was as long as 2.5 h for the study team. Second, the lack of electricity in most villages included in the study forced the campaigns to take place during daylight. To adapt the study as much as possible to the real-life setting of the daily work routine at health centers, campaigns were not held on weekends. In order to minimize the effect of individual team members on the outcomes, the teams rotated between the study-arms—meaning that one team ran campaigns in the HB-HTC group in one week and in the MC-HTC group the next week, and so on.

### Intervention

The MC-HTC group provided services through “pitso”—the more routine HTC approach in Lesotho. “Pitso” is a community gathering, usually held at the chief's place. One week prior to the scheduled date of the campaign, the health centers informed the villagers via their chiefs about the planned community gathering with provision of HTC and other services. The day of the campaign, community members gathered to listen to a health talk given by a primary health care nurse aimed at sensitizing listeners to HIV as well as other health issues. After the talk, all participants of the community gathering were invited to access the services in one of two tents installed next to the chief's place. Persons interested queued in front of the tent, entering one by one to receive the sought services. Families and couples were allowed to enter the tent together to receive services simultaneously, if they wished. Inside the tent, one lay counselor and one nurse offered all services of the multi-disease campaign as listed above. The participant was free to accept or refuse any of the offered services. For example, a person was able to enter the tent and accept blood pressure and blood glucose measurement, but refuse HTC and all other services.

Identically to the MC-HTC group, villagers in villages assigned to the HB-HTC group were informed about the campaign 1 wk prior to the scheduled date. However, in the HB-HTC group there was no community gathering or health talk. After arrival at the village and a formal visit to the chief, the two sub-teams—each consisting of one lay counselor and one nurse—started from the chief's place and provided all the services at people's homes, going door-to-door towards the periphery of the village. Services were usually provided to all persons living in the same household at the same time. If nobody was at home, the teams moved to the next house. Identical to the MC-HTC approach, individuals were free to choose the services they wished to receive out of the offered package of the multi-disease campaign.

During both intervention approaches, a professional counselor supervised the two sub-teams. The sub-teams called the professional counselor in case of technical difficulties or if they encountered particular psycho-social problems during or after testing. Participants received no incentives, neither for participating in the study nor for accepting HTC.

### Eligibility of Study Participants

All persons resident within the study area and not known to be HIV-positive were eligible. Children <18 y who were not accompanied by their caregiver to provide consent for participation in the study were excluded. Persons with a documented last negative HIV test dated less than 12 wk ago were excluded from HTC, as national HTC guidelines recommend a 12-wk period before re-testing after a negative HIV test. Ethics approval was obtained from the National Research and Ethics Committee of the Ministry of Health and Social Welfare of Lesotho. Participants gave written and oral consent for their data to be analyzed anonymously and could exit the study at any point while continuing to access and receive the same services. Data were kept and processed confidentially.

### Outcomes at the Individual Level

The trial had three primary and five secondary outcomes. The first primary outcome was the number of persons taking up HTC. All eligible individuals accessing the multi-disease campaigns were offered HTC. Uptake was defined as acceptance and completion of HTC with pre-test counseling, HIV test, and post-test counseling. The second primary outcome was the proportion of participants newly tested HIV-positive among participants who took up HTC. Participants who reported that they had never received a positive HIV test result prior to the planned HTC and who tested subsequently HIV-positive were defined as newly tested HIV-positive. The third primary outcome was linkage to care within 28 d after a positive HIV test among participants who tested HIV-positive during the campaign. Participants who tested positive were referred to their nearest health center. One month later, the study team checked the pre-ART and ART registers of the facilities in the study area to see whether individuals had linked to chronic care within 28 d.

The first secondary outcome was the age group distribution of persons accessing the campaigns (<12, 12–24, and ≥25 y). The second secondary outcome was the proportion of first-time testers among people who took up HTC. “First-time tester” was defined as an individual reporting never having had an HIV test before and taking up HTC during the campaigns. The third secondary outcome was male participation. This outcome is reported in two ways: the proportion of men among all individuals who accessed the campaigns, and the proportion of men who took up HTC during the campaigns among all persons who accessed the campaigns. The fourth secondary outcome was the clinical WHO stage of participants newly diagnosed HIV-positive, as assessed by the study nurse during the campaign. The fifth secondary outcome was the CD4 cell count among participants newly diagnosed HIV-positive, measured on site with a point-of-care CD4 test (Alere Pima). With the exception of the third primary outcome, the nurse of each sub-team collected all data during the campaign, using a paper-based reporting form. Correct filling-in of the reporting form was described in standardized procedures. The nurses had been trained during four 1-d HTC campaigns prior to the study. Outcomes for other services provided during the multi-disease campaigns will be reported separately.

### Outcomes at the Cluster Level

One month after the end of the five campaigns per cluster, the study team assessed the number of persons who tested HIV-positive through routine facility-based HTC during the 28 d following the campaign in the specific cluster by reviewing the HTC register of the respective facility. The rationale was to identify whether a larger number of individuals—especially those at risk of HIV infection—sought HTC at their clinic as a result of sensitization through the campaigns. Aggregate data at the cluster level were collected separately for individuals residing in one of the five control villages who tested HIV-positive through routine HTC and overall individuals who tested positive within the cluster.

### Post Hoc Cost Analysis

In Table S1, we report the costs related to the 60 campaigns held during the study. Costs are reported based on the actual expenditures made for each component of the campaign. The initial budget was in maloti (LSL) and then transformed into US dollars using the exchange rate of September 2011 (1 LSL = US$0.12). Amortization of the two 4×4 vehicles used during the campaign was budgeted as being 8.5 LSL (US$1.02) per kilometer, based on real costs. Cost analysis was not part of the initial trial protocol.

### Sample Size and Statistical Analysis

The trial was designed and powered to measure outcomes at the individual level. The sample size was determined by the formula proposed by Hayes and Bennett [28]. From the experience of former HTC campaigns using the MC-HTC approach, we estimated that HTC uptake would be about 65% in the MC-HTC arm. The estimated difference was anticipated to be 15% (10% to 20%) in favor of HB-HTC. With an expected inter-cluster coefficient *k* of 0.1, six clusters per arm with 200 individuals per cluster would be needed to achieve a statistical power of 80% at a significance level of 5%. After completion of the trial, the calculated inter-cluster coefficient *k* was 0.04 for the first primary outcome. For the second primary outcome (newly detected HIV infections), *k* was close to zero, indicating that there was no relevant inter-cluster variance. Considering the measured difference in HIV prevalence in both arms, post hoc sample size computation for the second primary outcome indicated that 192 individuals per cluster were needed to achieve a statistical power of 80%. Analyses were run on STATA 12.1 (StataCorp).

All reported adjusted odds ratios (aORs) take into account a potential correlation between the estimated errors within the same cluster (*cl* option in STATA). In addition, all aORs and *p*-values are adjusted for gender and age group (<12 y, 12–24 y, and ≥25 y)—except for analyses stratified by age group or gender, where adjustment is done only for gender and cluster or age group and cluster, respectively. Because the national HTC guidelines of Lesotho require consent of the caregiver to test children <12 y of age, a separate analysis was performed for the first primary outcome, disaggregated by age group (distinguishing between children <12 y, adolescents and young adults 12–24 y, and adults ≥25 y). Median CD4 values from participants newly diagnosed as HIV-positive were compared using the Mann-Whitney U test. Outcomes from aggregate data at the cluster level are reported descriptively.

### Deviations from Initial Trial Registration

Aggregate data from clusters on numbers of persons newly tested HIV-positive through routine facility-based HTC during the 28 d following the study campaigns were initially registered as one of the primary outcomes. While finalizing the internal study protocol, cluster data were dropped as a primary outcome because the trial was designed and powered to measure differences in outcomes at the individual level and not at the cluster level. Only individual endpoints were kept as primary outcomes—uptake of HTC, participants newly detected HIV-positive, and subsequent enrollment in HIV care—as these endpoints represent the three major steps of the HTC cascade. Nevertheless, description of cluster data was maintained and is reported in this paper.

## Results

### Enrollment and Participants

**Figure 1:**
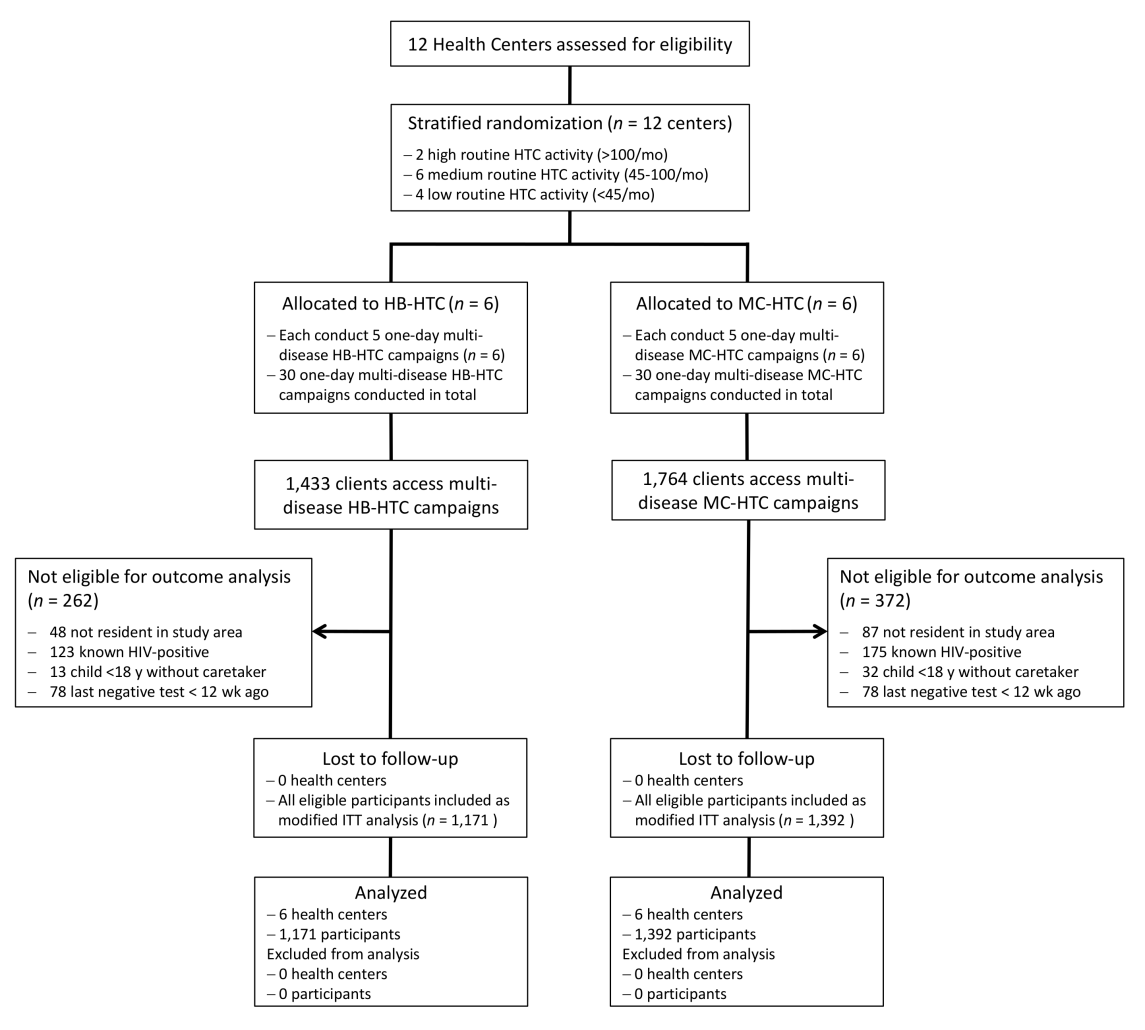
Consort chart of the study. One health center with its catchment area formed a cluster. ITT, intention to treat.

Characteristics of randomized clusters are displayed in Table 1. Figure 1 shows the enrollment, exclusion, and flow of the patients in both study arms. The HB-HTC campaigns provided services to 1,433 individuals, and the MC-HTC campaigns to 1,764 individuals. Overall, 634 individuals were excluded from the analysis due to the predefined exclusion criteria, 262 (18.3%) in the HB-HTC group, and 372 (21.1%) in the MC-HTC group. The main reason for exclusion was an already known positive HIV status. All eligible persons consented to their data being included in the analysis.

**Table 1:** Characteristics of randomized clusters and baseline characteristics of study participants.

Characteristic	HB-HTC Group (*n* = 6)	MC-HTC Group (*n* = 6)
**Median (IQR) catchment population per cluster**	6,311 (5,379–7,203)	4,909 (4,267–5,307)
**Median (IQR) routine facility-based HTC per cluster in the 3 mo prior to the study period (Jul–Sept 2011)**		
Men	42 (35–76.8)	39.5 (31–45.9)
Women	116 (101.5–125.8)	120 (75.8–135.8)
Both sexes	183.5 (140–207.1)	157 (111–164.5)
**Median (IQR) number of facility-based positive HIV tests per cluster in the 3 mo prior to the study period (Jul–Sept 2011)** ¶	12.5 (11.3–12.9)	11.5 (11–11.9)
**Number of study participants**	1,171	1,392
**Age group** §		
<12 y	296 (25.3%)	356 (25.6%)
12–24 y	208 (17.8%)	207 (14.9%)
≥25 y	658 (56.2%)	827 (59.4%)
Age not recorded	9 (0.8%)	2 (0.1%)
**Gender** *		
Women	776 (66.3%)	976 (70.1%)
Gender not recorded	4 (0.3%)	2 (0.1%)

¶ Data derived from national HTC registers of the 12 health centers in the study area.

§ *p*-Value for age group distribution: 0.88.

*p-Value for gender distribution: 0.044.

IQR, interquartile range.

### HTC Uptake, Positive Tests, and Linkage to Care

Table 2 displays the three primary outcomes. Overall, the HB-HTC group achieved a higher uptake of HTC (92.5% versus 86.7%; aOR: 2.06; 95% CI: 1.18–3.60; *p* = 0.011). There was a significant interaction between age and uptake of HTC (*p*<0.001): among children below 12 y of age—where the caregiver has to approve HTC—uptake was 87.5% in the HB-HTC group, compared to 58.7% in MC-HTC group (aOR: 4.91; 95% CI: 2.41–10.00; *p*<0.001). Among participants ≥12 y of age, uptake tended to be lower in the HB-HTC group compared to the MC-HTC group (Table 2). Out of the 2,563 participants in both groups, 114 (4.4%) tested HIV-positive. Participants in the HB-HTC group were less likely than participants the MC-HTC group to test positive: 39 (3.6%) versus 75 (6.2%) (aOR: 0.64; 95% CI: 0.48–0.86; *p* = 0.002) (Table 2).

**Table 2:** Primary outcomes.

Outcome	Sub-Catetgory	HB-HTC Group, Percent (*n/N*)	MC-HTC Group, Percent (*n/N*)	OR (95% CI)	*p*-Value	aOR (95% CI)	Adjusted *p*-Value
**Uptake of HTC**	**Overall**	92.5 (1,083/1,171)	86.7 (1,207/1,392)	1.89 (1.44–2.46)	<0.001	2.06 (1.18–3.60)	0.011
	**By age group**						
	<12 y	87.5 (259/296)	58.7 (209/356)	4.92 (3.29–7.37)	<0.001	4.91 (2.41–10.00)	<0.001
	12–24 y	94.7 (197/208)	97.1 (201/207)	0.53 (0.19–1.47)	0.226	0.59 (0.21–1.67)	0.322
	≥25 y	93.9 (618/658)	96.1 (795/827)	0.62 (0.39–1.00)	0.051	0.61 (0.33–1.13)	0.117
	**By gender**						
	Men (≥12 y)	94.3 (247/262)	97.1 (236/243)	0.49 (0.19–1.22)	0.125	0.49 (0.18–1.29)	0.149
	Women (≥12 y)	94.2 (565/600)	96.1 (758/789)	0.66 (0.40–1.08)	0.101	0.65 (0.38–1.11)	0.116
**Participants newly detected HIV-positive**		3.6 (39/1,083)	6.2 (75/1,207)	0.56 (0.38–0.84)	0.005	0.64 (0.48–0.86)	0.002
**Linkage to care after positive HIV test**		25.6 (10/39)	25.3 (19/75)	1.02 (0.42–2.47)	0.971	0.99 (0.35–2.79)	0.978

All aORs and adjusted *p*-values are adjusted for cluster effect. In non-stratified analyses, aORs and *p*-values are also adjusted for gender and age group. Missing data for gender: 6; missing data for age group: 11.

OR, odds ratio.

Pre-ART and ART registers of the 12 clusters were checked for all 114 patients who tested positive. Ten (25.6%) out of the 39 in the HB-HTC group and 19 (25.3%) out of the 75 in the MC-HTC group linked to HIV care at the nearest facility within 1 mo (Table 2).

### Age of Participants, First-Time Testers, and Participation of Men

The two groups had a similar age distribution (Table 1). Overall, the proportion of first-time testers was higher in the HB-HTC group (56.2% versus 44.4%; aOR: 1.57; 95% CI: 1.03–2.39; *p* = 0.035), in particular among adolescents and young adults aged 12 to 24 y (53.8% versus 31.3%; aOR: 2.47; 95% CI: 1.49–4.08; *p*<0.001) (Table 3). The proportion of men was 30.3% and 23.5% in the HB-HTC and MC-HTC groups, respectively. However, after adjusting for clustering, the difference was not significant anymore (aOR: 1.41; 95% CI: 0.98–2.03; *p* = 0.062) (Table 3).

**Table 3:** Proportion of first-time testers, participation of men, and proportion of participants newly tested HIV-positive with advanced disease.

Outcome	Sub-Category	HB-HTC Group, Percent (*n/N*)	MC-HTC Group, Percent (*n/N*)	OR (95% CI)	*p*-Value	aOR (95% CI)	Adjusted *p*-Value
**Proportion of first-time testers among participants who took up HTC**	**Overall**	56.2 (609/1,083)	44.4 (536/1.207)	1.61 (1.36–1.89)	<0.001	1.57 (1.03–2.39)	0.035
	**By age group**						
	<12 y	89.2 (231/259)	76.1 (159/209)	2.59 (1.57–4.29)	<0.001	2.64 (0.76–9.24)	0.128
	12 to 24 y	53.8 (106/197)	31.3 (63/201)	2.55 (1.69–3.84)	<0.001	2.47 (1.49–4.08)	<0.001
	≥25 y	43.2 (267/618)	39.4 (313/795)	1.17 (0.95–1.45)	0.146	1.15 (0.73–1.79)	0.552
**Participation of men**	Proportion of men among individuals (≥12 y) reached through the campaigns	30.3 (262/866)	23.5 (243/1,034)	1.42 (1.16–1.74)	0.001	1.41 (0.98–2.03)	0.062
	Proportion of men who took up HTC among individuals (≥12 y) reached through the campaigns	28.5 (247/866)	22.8 (236/1034)	1.35 (1.10–1.67)	0.004	1.35 (0.92–1.99)	0.129
**Proportion of participants newly tested HIV-positive with clinical WHO stage 3 or 4 disease**		5.1 (2/39)	1.3 (1/75)	NA	NA	NA	NA

All aORs and adjusted *p*-values are adjusted for cluster effect. In non-stratified analyses, aORs and *p*-values are also adjusted for gender and age group. Missing data for gender: 6; missing data for age group: 11.

NA, not applicable; OR, odds ratio.

### Stage of Disease among Participants Newly Tested HIV-Positive

Median CD4 count (interquartile range) among participants newly tested HIV-positive was 438 cells/µl (265–650) in the HB-HTC group and 400 cells/µl (207–629) in the MC-HTC group (*p* = 0.491); 39% and 35% had a CD4 count <350 cells/µl, respectively (aOR 0.84; 95% CI: 0.28–2.55). Only one participant in the MC-HTC group and two in the HB-HTC group had a clinical WHO stage 3 infection. The remaining participants were staged as WHO stage 1 or 2 (Table 3).

### Outcomes at the Cluster Level

During the 28 d that followed the campaigns, 49 individuals tested HIV-positive in the 12 clusters by visiting routine facility-based HTC. Seven of those resided in the predefined control villages of the study (six in control villages of the HB-HTC intervention and one in a control village of the MC-HTC intervention). Within the HB-HTC and MC-HTC clusters, 12 and 30 individuals, respectively, were identified as HIV-positive but resided outside the study villages (intervention and control).

### Cost of Campaigns

Table S1 summarizes the expenditures corresponding to the 30 1-d HTC campaigns in each arm. Respective costs in the HB-HTC and MC-HTC arms were US$17.8 and US$16.2 per individual who took up HTC, US$495 and US$260 per individual who tested HIV-positive, and US$1,932 and US$1,027 per person who tested HIV-positive and enrolled in care within 1 mo.

## Discussion

This cluster-randomized trial compared two ways of providing community-based HTC in rural Lesotho: the approach of community gathering and subsequent HTC in a mobile clinic (MC-HTC) and the approach of going from door to door and offering HB-HTC to all household members. The trial was powered to show a difference in HTC uptake of 15% or more between the two arms. HB-HTC and MC-HTC had similar uptake of HTC for adolescents and adults ≥12 y, but HB-HTC achieved a significantly higher uptake in children below 12 y of age. On the other hand, MC-HTC led to more persons being newly detected as HIV-positive. In both study groups, there was high attrition of participants between receiving a positive HIV test result and linkage to care, with only one-quarter presenting at their local health facility within 1 mo after their positive test result. HB-HTC and MC-HTC differ in their sampling strategy and therefore reach different populations. HB-HTC reached more children, first-time testers, and men, whereas MC-HTC reached more individuals with a previously undiagnosed HIV infection.

The literature provides conclusive evidence that community-based HTC is a cost-effective approach to reach populations that have low access to clinic-based HTC, results in higher uptake of HTC, and detects more new HIV infections at an asymptomatic stage [11],[12],[29]. Only a few observational studies, however, have compared different approaches of community-based HTC. Our study therefore adds to the limited evidence.

The study has several limitations. First, available resources limited the number of clusters included in the study, which naturally resulted in high standard errors and broad confidence intervals as all outcomes were adjusted for clustering. Second, based on routine HTC data in the study area (Table 1) and on the high overall HIV prevalence in Lesotho, we had assumed a higher proportion of positive HIV test results among study participants. However, prevalence in the study population turned out to be lower. Although this may partly be explained by a relatively high number of individuals accessing the multi-disease campaigns who had to be excluded because they were already known to be HIV-positive, addition of these individuals results in a prevalence still below expected. This indicates that the campaigns may have reached an underexposed population. Several observational studies from southern Africa report lower HIV prevalence among testers during mobile testing campaigns than in clinic testing [30]–[35]. However, this should not draw into question the value of community-based approaches to HTC. Knowing one's HIV status—irrespective if positive or negative—carries an important preventive benefit for the individual as well as for the community [4]. Another important reason for the low rate of positive HIV tests in this study may be the limitation of campaigns to a time frame from 9:00 a.m. to 5:00 p.m. during weekdays. Campaigns thus did not reach persons working outside the village, and in this case excluded migrant workers who work in neighboring South Africa—a group known to be at particularly high risk of HIV infection [36]. To reach working populations and children or adolescents who are at school, HTC campaigns should either be extended to evenings and weekends or to workplaces and schools [12]. Third, the study did not assess reasons for not linking to care among persons newly tested HIV-positive who did not enroll in care within 1 mo. Several studies have shown that interventions such as follow-up visits or phone calls may improve linkage to care [37],[38]. Fourth, our study was not designed to collect follow-up data on persons newly diagnosed HIV-positive to assess possible harms as a consequence of knowing their positive HIV status. There is a risk of gender-based violence, stigma, or coercion as a result of a positive HIV test [14],[39],[40]. In its consolidated ART guidelines of 2013, WHO reported on the findings of 15 studies that examined positive and negative consequences of community-based HIV testing. The reviewed evidence demonstrated neither reduction nor increase in stigma, fear, or harm through community-based approaches [40]. During our study, lay counselors were closely supervised by the professional counselor, and direct individual feedback of the test result—an important ethical part of any broad HTC activity [41]—was ensured as an integrated part of the campaigns. All patients testing positive during the campaigns were followed up by a lay counselor or a village health worker if they did not link to care within 1 mo. Fifth, the exclusion criteria in both study groups led to the exclusion of about one-fifth of persons accessing the campaigns (Figure 1). About half of exclusions were due to an already known HIV status. Another important reason for exclusion was persons who stated that they had their last negative HIV test <12 wk ago.

The trial was designed and set up to reduce potential bias as much as possible within a setting that resembles real conditions and, as such, allows practical conclusions. Inherently to such setting, we cannot fully rule out that reporting and recording bias may have occurred. Under the assumption that behavior and perceptions did not vary between the different groups and clusters, reporting bias—if present—can be assumed to have occurred equally distributed between groups. Rotation of the teams between groups and clusters also reduced the risk of the impact of recording bias on the final result and conclusion. Although the villages where the campaigns were conducted were selected according to the same criteria within each cluster, a selection bias cannot be excluded. Equally, HB-HTC cannot exclude selection bias of individual households and persons, given the non-random selection of households within one village. Nevertheless, we had decided not to use random sampling of households in HB-HTC, as MC-HTC does not represent a random sampling either, given that persons have to decide to attend the community gathering. As the interventions could not be concealed, a potential detection bias cannot be excluded. It is important to realize that the interventions reached different sub-populations. Enrollment of participants was inherently done within a different recruitment context (HB-HTC versus MC-HTC) and different conditions. The MC-HTC arm reached persons who had decided to access the campaign, whereas HB-HTC reached individuals who were at home while the campaign was held. This difference was evident in imbalances in the characteristics of the participants of the two groups. Such imbalances have been said to challenge the principles of intention to treat analysis in cluster-randomized trials [42]. Nevertheless, one of the study's objectives was exactly to assess differences in populations met by the two approaches: from a programmatic point of view, the information about “which intervention reaches what kind of population” is very important. In addition, some clusters were geographically neighbored, and, as a consequence, inter-cluster cross-contamination related to aggregate outcome data at the cluster level 28 d after the campaigns might have occurred.

To our knowledge, this is the first trial comparing the efficacy of HB-HTC to that of community-gathering HTC around a mobile clinic. Apart from the real-life setting of the study, the additional strength of our study was that it tested both approaches within a standardized multi-disease campaign setting with equal use of resources. Furthermore, the study assessed as outcomes not only HTC uptake but also persons newly detected as HIV-positive and their subsequent enrollment in care.

Both study arms demonstrated a high HTC uptake of >85%. At 92.5%, uptake in the HB-HTC group was higher than reported in the systematic review of community-based HTC approaches by WHO that calculated an average uptake of 87% for MC-HTC and 80% for HB-HTC [12]. The higher uptake in our study may be due to the provision of HTC within a multi-disease campaign, offering clinical tuberculosis screening and measurement of weight and height for all individuals, family planning, blood pressure and blood sugar measurement for adults, and provision of vitamin A, standard immunizations, and deworming for eligible children and women. This multi-disease campaign approach may have helped to reduce the stigma related to HTC, as HTC was only one part of a broader package of services.

HTC uptake in the HB-HTC group (92.5%) was significantly higher than in the MC-HTC group (86.7%). This overall difference was due to a much higher uptake in the age group <12 y in the HB-HTC group (Table 2). Interestingly, the proportion of children <12 y accessing the campaigns was the same in both groups (Table 1). However, only 59% of childrens' caregivers in the MC-HTC group accepted HTC as compared to 88% in the HB-HTC group (Table 2). This result implies that caregivers' consent for the child's HTC is more easily received within the home than at a mobile clinic.

HB-HTC reached more men for HTC. However, after adjustment for cluster effect, the difference was not significant anymore. Previous studies have reported that men are reluctant to seek group testing in general and suggest that confidential testing at home may be a better approach to reach this underrepresented group for HTC [34],[35],[43]. Furthermore, HB-HTC reached more first-time testers, particularly among adolescents and young adults. Given the need to scale up HTC—especially among men, first-time testers, and children [2],[43],[44]—our results suggest that HB-HTC may prove a good approach to reach these population groups.

The prevalence of previously undetected HIV infections was lower in the HB-HTC group. While the MC-HTC approach may inherently attract individuals already concerned about their health, a possible second cause may be the effect of the health talk during community gatherings on undecided individuals. Participants may reflect on the information received and make a decision to test or not according to their personal judgment of their risk profile, leading to participants with a higher pre-test probability of infection taking up HTC in the MC-HTC group. If the goal of an HTC campaign is to maximize the number of individuals newly diagnosed HIV-positive to consecutively enroll into care, the community-gathering approach around a mobile clinic may prove more effective.

Only one out of four participants newly diagnosed HIV-positive linked to HIV care within 1 mo. Reasons for the particularly low linkage in our study may be the rural and remote context, where transport is a major barrier to accessing care [45]. A time period of 3 mo, used in other studies conducted in rural areas, to assess linkage to care might have resulted in a higher rate [45]. Moreover, most newly diagnosed persons in our study were at an oligo-symptomatic stage of disease and had a CD4 count >350 cells/µl. Less advanced stage was associated with low linkage to care in South Africa and Uganda [38],[45]. Two recent observational studies in South Africa reported low linkage to care after testing at a mobile clinic. Basset and colleagues found that within 3 mo, only 10% linked to care in a township in Durban, whereas Govindasamy et al. reported 51% having linked to care within the predefined period in peri-urban townships in Cape Town [32],[33]. These studies, as well as our findings, suggest that mobile HTC campaigns—be it MC-HTC or HB-HTC—may have a limited effect on timely linkage to care of previously undiagnosed HIV-positive persons, unless testing is conducted in combination with effective interventions to improve linkage to care. Several trials on interventions to improve linkage to care are still ongoing. Approaches using intensified peer support, incentives, home-based ART initiation, or short message service or phone call reminders appear to be promising interventions [46].

### Conclusion

Overall, both study groups achieved a high uptake of HTC, endorsing the use of community-based multi-disease approaches to reach populations that may not access facility-based HTC. Based on our results, the choice between HB-HTC and MC-HTC should be guided by the intervention's objective. HB-HTC may be a preferred option where equity in access to HTC is of concern, or in a new area to increase coverage of HTC and reach unreached individuals and groups, such as first-time testers, children, and men. For routine activities, however, the MC-HTC approach may prove more appropriate, especially when detection of new HIV infections is the major goal. In the light of the recent 90-90-90 strategy [47], HB-HTC appears to be a promising approach to improve coverage of hard-to-reach populations, such as children, first-time testers, and men—however, only if combined with effective interventions improving linkage to care.

## Supporting Information

Table S1 Expenses for the 30 1-d HTC campaigns per study arm.
(DOCX)

Text S1 CONSORT table.
(DOC)

Text S2 Trial protocol.
(DOCX)

## Abbreviations

aOR: adjusted odds ratio
ART: antiretroviral therapy
HB-HTC: home-based HIV testing and counseling
HTC: HIV testing and counseling
LSL: maloti
MC-HTC: mobile clinic HIV testing and counseling
