# Supplementary material for: Home-Based Versus Mobile Clinic HIV Testing and Counseling in Rural Lesotho: A Cluster-Randomized Trial
Source: PLoS Med. 2014 Dec 16;11(12):e1001768. doi: 10.1371/journal.pmed.1001768 (PMC4267810; doi:10.1371/journal.pmed.1001768)
Supplement: Table S1 — Expenses for the 30 1-d HTC campaigns per study arm. (DOCX) [file pmed.1001768.s001.docx]

**Table S1 – Expenses made for the 30 one-day HTC-campaigns per study-arm**

|  |  | | | **HB-HTC** | | | **MC-HTC** | |
| --- | --- | --- | --- | --- | --- | --- | --- | --- |
| **Item** | **Cost per item (USD)** | | | **Quantity** | | **Cost (USD)** | **Quantity** | **Cost (USD)** |
| Nurses’ salaries (4 nurses, 2 months) | 960 per month | | | 4 | | 3840 | 4 | 3840 |
| Lay counsellors’ salaries (4 lay counsellors, 2 months) | 150 per month | | | 4 | | 600 | 4 | 600 |
| Professional counsellors' salaries (2 professional counsellors, 2 months) | 720 per month | | | 2 | | 1440 | 2 | 1440 |
| Drivers’ salaries (2 drivers, 2 months | 300 per month | | | 2 | | 600 | 2 | 600 |
| Amortisation of 2 4x4 vehicles | 1.02 per km | | | 3,000 | | 3,060 | 3,000 | 3,060 |
| Alere Determine test-kits | 1.02 per test | | | 1,083 | | 1,105 | 1,207 | 1,231 |
| Alere Double Check Gold test-kits | 1.92 per test | | | 39 | | 75 | 75 | 144 |
| Point-of-care CD4-counter (including 100 cartridges) | 7,218 | | | 1 | | 7,218 | 1 | 7,218 |
| Other equipment (gloves, lancets, gazes, tents) |  | | |  | | 480 |  | 480 |
| Staff accomodation, perdiems, horse-rent |  | | |  | | 900 |  | 900 |
| **Total cost** |  |  |  | | **19,318** | |  | **19,513** |
