# Supplementary material for: Home-Based Versus Mobile Clinic HIV Testing and Counseling in Rural Lesotho: A Cluster-Randomized Trial
Source: PLoS Med. 2014 Dec 16;11(12):e1001768. doi: 10.1371/journal.pmed.1001768 (PMC4267810; doi:10.1371/journal.pmed.1001768)
Supplement: Text S2 — Trial protocol. (DOCX) [file pmed.1001768.s003.docx]

**Comparison of door-to-door versus community gathering approach in delivering an integrated package of services to the community –**

**a cluster randomized study**

**Internal Protocol on procedures and analysis for all persons involved in the conduction of the study**

**Author:**

Niklaus Labhardt

**Commented and approved by:**

Masetsibi Motlomelo

Kamele Mashaete

Jochen Ehmer

Karolin Pfeiffer

Michael Hobbins

**Contacts**:

[n.labhardt@solidarmed.ch](mailto:n.labhardt@solidarmed.ch)

# Table of contents

Table of contents 2

Aim of the protocol 3

Partners and persons responsible in the field 3

Overall responsibility 3

Partners 3

Persons responsible for implementation of campaigns: 3

Persons responsible for data-collection and processing: 3

On site study-supervisors: 3

Explanation of abbreviations and terms: 3

Study-Methods 4

AIM OF THE STUDY 4

Hypothesis 4

OBJECTIVES OF THE STUDY 4

STUDY DESIGN 5

STUDY SETTING 5

INTERVENTIONS 5

Randomization and allocation 6

Conduction of campaigns 7

Eligibility criteria 7

Exclusion criteria 7

Selection of villages, households and individuals 8

Outcomes measured 9

Sample size 11

COLLECTED VARIABLES AND THEIR ANALYSIS 12

Statistical Analysis 13

Selection biases and confounders 13

Study-flow 14

Ethical Considerations: 15

Facilities and teams conducting testing campaigns 15

Informed consent 15

Compensation 15

Confidentiality 15

Annex 1: Standards of procedures 16

Annex 2: Study-questionnaire (data-collection tool) 19

Annex 3: Informed Consent form 20

# Aim of the protocol

This protocol shall serve as an internal memorandum on methods and procedures of the Door-to-Door versus Pitso-study to be held from October to December 2011 within the project area of the SolidarMed project SMART III in Lesotho. It is based on the proposal submitted to the National Ethics Committee but elaborates more in detail the operating procedures. It is addressed to all staff – either at headquarters SolidarMed in Switzerland or in the field in Lesotho in order to ensure a common understanding of the study and consistency in study-procedures.

# Partners and persons responsible in the field

## Overall responsibility

‘Me Masetsibi (SolidarMed) and Niklaus Labhardt (SolidarMed)

## Partners

**Primary Health care team Paray:** Represented by ‘Me Matefo (PHC-coordinator) and ‘Me Mpho (HIV/AIDS coordinator)

**Primary Health care team Seboche:** Represented by ‘Me Mokhantso (PHC director) and ‘Me Matseliso (HIV/AIDS coordinator)

**District Health Management team Thaba-Tseka:** Represented by ‘Me Moshoeshoe (senior counselor)

**District Health Management team Botha-Bothe:** ‘Me Manquosa (senior counselor)

## Persons responsible for implementation of campaigns:

**Seboche:** ‘Me Mokhantso (PHC-director) and ‘Me Kamele (professional counselor)

**Paray:** ‘Me Matefo (PHC-director)

## Persons responsible for data-collection and processing:

**Seboche:** ‘Me Masetsibi (site-coordinator SolidarMed Seboche) assisted by ‘Me Kamele

**Paray:** Ntate Khotso (operational research assistant SolidarMed)

## On site study-supervisors:

‘Me Kamele, ‘Me Masetsibi, Ntate Khotso

# Explanation of abbreviations and terms:

**HTC:** HIV Testing and Counselling

**ART:** Antiretroviral Treatment

**Pitso**: Traditional way of community gathering to promote health services. In the case of promotion of HTC, the community is mobilized through village-authorities (chief) a few days prior and then gathers near the chief’s place. After a general health education to all people attending, services are provided within two tents (mobile clinic) that ensure confidentiality.

**Door-to-door**: After having been sensitized through village-authorities a few days ago (same way of sensitization as for pitso), health care workers visit the people’s homes where they propose the services (“home-based testing”).

# Study-Methods

## AIM OF THE STUDY

To compare door-to-door versus pitso in delivering HTC within an integrated package in rural Lesotho in terms of people newly tested positive for HIV and enrolled into chronic HIV-care.

## Hypothesis

In comparison to the traditional “Pitso”-approach (community gathering), the door-to-door approach will result in a higher uptake of HTC, more newly detected HIV-positive persons and more patients newly enrolled into chronic HIV/AIDS-care using the same resources (financial, time and HR). The effect will be measurable as a a higher proportion and absolute number of people being tested HIV-positive, and on a cluster-level (health center) by a higher number of people enrolled in chronic HIV/AIDS care during the month following the campaigns.

## OBJECTIVES OF THE STUDY

1. Main objective
   1. To compare door-to-door versus pitso in terms of:
      1. HTC-uptake
      2. Newly tested HIV-positive persons
      3. Enrollment into chronic HIV/AIDS care (pre-ART or ART) among newly tested HIV-positive persons
2. Secondary objectives
   1. To compare door-to-door versus pitso in terms of
      1. Proportion of first-time testers
      2. Patient-characteristics (age, sex, first-time tester)
      3. Average CD4-count among clients newly tested HIV-positive
      4. Clinical WHO-stage among clients newly tested HIV-positive
      5. Uptake of other services provided
         1. Blood-pressure measurement
         2. Blood-glucose measurement
         3. Family Planning
         4. Immunization
         5. Deworming
         6. Vitamin A
      6. Detection of Tuberculosis suspects
      7. Sputum turn-around of Tuberculosis suspects
      8. Sputum-results of Tuberculosis suspects
   2. To assess among the clients participating in the study the rate of:
      1. High Random Glucose
      2. High Blood Pressure
      3. Undernutrition/Obesity

## STUDY DESIGN

Prospective cluster-randomized study. Each health center within the SMART project area (n=12) with its catchment area represents a cluster. One study arm provides integrated HTC using the door-to-door approach (6 HCs) as compared to the other arm using the pitso-approach (6 HCs)

## STUDY SETTING

The study will take place within the catchment areas of Seboche and Paray Mission hospital. Both hospitals belong to the Christian Health Association of Lesotho (CHAL). Seboche hospital is situated in the northern Lesotho, district Butha Buthe, has an estimated population of about 55’000 and an estimated adult HIV-prevalence of 16%. Paray Mission hospital is situated in the central district Thaba Tseka with an estimated catchment population of 77’000 and an estimated HIV-prevalence of 20%. Routine ART and HTC services are offered at the hospitals and at the health centres belonging to the catchment areas (5 health centres in Seboche hospital and 7 in Paray hospital catchment area). Under the supervision of the professional and senior counselor, lay-counsellors perform HTC at hospital as well as health center level. The catchment populations of health centers vary between 4’000 and 10’000 in 20 to 60 main-villages that are sometimes again divided into up to 5 sub-villages.

## INTERVENTIONS

### Campaigns

In both study-arms campaigns promoting HTC within an integrated package of primary health care services are held. One study arm promotes the package using the pitso approach whereas the other arm applies the door-to-door approach. The mobilization of the community is done 4 to 7 days prior to the campaign using the same means (village authorities) in both approaches. For both approaches mobilization is done through a letter to the village chief where the activity and the date are announced. The village chief has then the responsibility to inform the community (this is an often applied approach and all village chiefs are used to this procedure). Teams consisting of 2 nurses, 2 lay-counselors and one study-supervisor, will perform the campaigns for the integrated package. The campaigns are held during five days, Monday to friday (each day another village) in 5 selected villages in the catchment area of each of the 12 health centers. If there are enough human resources at the health center, 1 nurse and 1 lay-counselor will be delegated from the health center, the other nurse and lay-counsellor are from the primary health care team of the hospitals (Seboche or Paray) in order to ensure consistency in procedures. In case of shortage at health center level, the whole team will be delegated from the hospital.

In both kind of campaigns exactly the same service package is provided. It consists of:

1. HIV counseling and testing
2. Blood Sugar testing
3. Blood Pressure measurement
4. Tuberculosis screening
5. Immunization for eligible children and women
6. Vitamin A for eligible children and women
7. Deworming for eligible children
8. Family planning for eligible women
9. Health Education focusing on HIV/AIDS and TB for all

### Procedures

The procedures are defined in the Standard of Procedures (SOP) manual (see annex 1). Study-team-members have been instructed on the SOPs and conducted 4 pre-test campaigns under supervision. The pre-test villages belong to catchment areas that are not part of the study.

In case the team encounters severely ill clients with danger signs, the patients will be transported in the study-car to the hospital (Seboche or Paray hospital), see annex 1.

### Intervention 1: Community gathering HTC

In case of intervention 1, the integrated package will be delivered through community gatherings (pitso). After mobilization through village authorities, the community will be sensitized in health talks and subsequently invited to get the integrated package in one of two tents that ensure confidentiality.

### Intervention 2: Door-to-door HTC

In case of intervention 2, the integrated package will be proposed at home. After information of the community via village authorities, two teams (each consisting of one lay-counselor and one nurse) will visit the people’s homes and propose the integrated package to the families.

## Randomization and allocation

### Clusters and sub-clusters

One Health Center with its catchment area forms a cluster. There are 12 Health Centers participating in the study, resulting in 6 Clusters per arm. The interventions (HTC-campains) will be held in 5 sub-clusters (villages) each.

### Randomization

The 12 health centers were openly randomized into two equal groups. Before randomization, clusters were stratified and paired according to their average-numbers of monthly new HIV-positive tested clients at routine clinic-based HTC (routine data from October to December 2010).

During an official meeting held on October the 3rd, a person not involved in the study threw a coin for one health center in each pair to allocate it either to the door-to-door or the pitso-arm. The other health center in the pair then automatically fell into the other arm. Table 1 shows the cluster-pairs and their allocation:

| **Pitso-arm** | **Door-to-Door arm** | **Time-frame of campaign** |
| --- | --- | --- |
| St Theresa HC (Paray) | St Peters HC (Seboche) | 17.-21. October 2011 |
| Ngoajane HC (Seboche) | Sehong Hong HC (Paray) | 24.-28. October 2011 |
| Linakeng HC (Paray) | Mokoto HC (Paray) | 31.10.-4.11. 2011 |
| Muela HC (Seboche) | Mohlanapeng HC (Paray) | 7.-12. November 2011 |
| Katse HC (Paray) | Boiketsiso HC (Seboche) | 14.-18. November 2011 |
| Khohlonso HC (Seboche) | Makhunoane HC (Seboche) | 21.-25. November 2011 |

Table 1 Matched pairs of clusters (health centers), their allocation to the study-arms and period where campaigns are held.

## Conduction of campaigns

Per cluster, campaigns are held during 5 days (Monday to Friday), each day in another village (subcluster) within the catchment area of the health center. Depending on the allocation of the cluster, either 5 days of door-to-door or 5 days of pitso campaigns are held. Both kinds of campaigns receive the same human and time-resources: 2 nurses, 2 lay-counsellors and one study-supervisor; timeframe: campaigns start at 9 am and end at 5 pm. A matched cluster-pair holds their campaigns at the same time (one pitso, the other door-to-door).

## Eligibility criteria

### Clusters

All Health Centers (12) within the SolidarMed SMART project area are eligible

### Sub-clusters (villages)

Villages are eligible if:

- clearly confined to the catchment area of the health center (not on border between two catchment areas
- Population of at least 25 households
- Distance to health center 3 to 20km

### Individuals

Individuals are eligible if:

- resident in the catchment area of the health center where the campaign is conducted
- Provision of written informed consent to participate (signed by writing or fingerprint) (see annex)
- In case of children (<16yrs): Provision of informed written consent by care-taker (signed by writing or fingerprint)
- Clients refusing consent for HTC but providing consent to participate in the study are still eligible

Eligibility for HTC:

- Eligibility for HTC follows the national HTC guidelines. All clients with an unknown HIV-status and who did not test negative within the previous 3 months, who are ≥ 12 years or are in company of an adult (≥18 years) care-taker who provides consent, are eligible. For HTC the client or his/her care-taker must provide written informed consent on the national HTC-consent form. In case of children below < 18 months, HTC is still offered as recommended in the national guidelines. However, in case of a positive test-result, referral to health center for PCR-testing is done.

Individuals who are not eligible for the study are nevertheless offered the same service-package.

## Exclusion criteria

### Sub-clusters (villages)

Villages are excluded from the study if:

- Not fulfilling inclusion criteria
- Village-authorities are opposed to the campaigns

### Individuals

Individuals are excluded from the study if:

- Not fulfilling eligibility criteria

In addition individuals are excluded for the analysis of the primary outcomes if:

- Already known HIV-positive
- Prior HIV-test < 3 months (window-period)

## Selection of villages, households and individuals

**Selection of communities:**

Before being informed if they are allocated to door-to-door or to pitso, the health center staff provides a list of ten villages within their catchment area that fulfill the eligibility criteria (see above) and that have not benefited from any sensitization or screening campaign in the past two years. From this list five villages will be randomly^[[1]](#footnote-1)^ chosen to receive the campaign (either door-to-door or pitso).

**Selection of households/place:**

- Door-to-Door approach: Within the villages the households are approached systematically starting from the chief’s place (moreneng), going to the periphery. A household is defined as one or several houses grouped together that are inhabited by one family. In case, a household is empty, the next household towards the periphery is chosen.
- Pitso approach: The pitso will be held at the place allocated by the village authorities, usually at the chief’s place (moreneng).

**Selection of individuals:**

- Door-to-Door: All members of a household (irrespective of age) will be proposed the integrated package including HTC.
- Pitso approach: All persons attending the pitso will be invited to receive the integrated package

## Outcomes measured

### Sources of outcomes

Sources of the different outcomes measured are the study-questionnaire (see annex 2), and the following official registers at the health centers: HTC-register, pre-ART register, ART-register, tuberculosis-suspect register

| *Outcome variable* | *Hypothesis* | *Source/Measure* | *Statistical Method* |
| --- | --- | --- | --- |
| **Primary outcomes** |  |  |  |
| 1. Proportion and absolute number of clients accepting HTC | Higher in door-to-door | Source: study-questionnaire | logistic regression |
| 1. Proportion and absolute number newly tested HIV-positive | Higher in door-to-door | Source: HIV-test result from Determine® & Double Check® (according national guidelines) [binary/count] | logistic regression |
| 1. Proportion and absolute number newly enrolled on chronic HIV/AIDS care within one month after the campaign of those newly tested during campaign | Higher in door-to-door | Source: study-questionnaire and pre-ART and ART-Registers. Enrolled (Y/N) [binary/count] | logistic regression |

| **Secondary outcomes** |  |  |  |
| --- | --- | --- | --- |
| 1. Proportion of first-time testers | Higher in door-to-door | Study-questionnaire [binary] | logistic regression |
| 1. Age of clients attending HTC | Lower in door-to-door | Study-questionnaire [cont] | t-test/ranksum test |
| 1. Sex of clients attending HTC | Proportion of male testers is higher in door-to-door | Study-questionnaire [binary] | logistic regression |
| 1. Proportion newly tested HIV-positive with advanced disease (WHO-stage 3 or 4) | - | Study-questionnaire [binary] | logistic regression |
| 1. Average CD4-count of clients newly tested HIV-positive | - | On-site CD4-count using PIMA^TM^ [cont] | t-test/ranksum-test |
| 1. Absolute number and proportion of clients screened positive for tuberculosis | - | Study-questionnaire [count/binary] | logistic regression |
| 1. Absolute number and proportion of clients positively screened for tuberculosis who bring their sputum to the facility within ≤ 5 days | - | Study-questionnaire and tuberculosis-suspect register | logistic regression |
| 1. Absolute number and proportion of clients bringing their sputum bottles who have a positive direct sputum smear | - | Study-questionnaire and tuberculosis-suspect register | logistic regression |
|  |  |  |  |

Table 2 Primary and secondary outcomes measured in the study

## Sample size

Expected difference in HTC-uptake between study-arms (1^st^ primary outcome): 80% (door-to-door) versus 65% (pitso)

k= 0.1

Power: 80%; Significance level: 5%

clients per cluster: 200 (persons attending the services per cluster)

According to formula of Hayes & Benett^[[2]](#footnote-2)^: 6 clusters per arm

## COLLECTED VARIABLES AND THEIR ANALYSIS

### Variables collected during testing-campaigns

| Variable | Type of variable |
| --- | --- |
| Age | Continuous |
| Sex | Binomial |
| Weight (kg) | Continuous |
| Height (cm) | Continuous |
| Accepts HIV-test (Y/N) | Binomial |
| 1^st^ time tester (Y/N/missing) | Binomial |
| Test result (Pos/Neg/Intermediate) | Categorical |
| Enrolled on pre-ART or ART within 1 month (Y/N) | Binomial |
| Absolute CD4-count the day tested positive | Continuous |
| Clinical WHO-stage the day tested positive | Ordinal |
| TB-screening (Pos/Neg/Missing) | Binomial |
| Blood-Pressure in mmHg | Continuous |
| Blood-Glucose in mmol/l | Continuous |
| Uptake of deworming and Vitamin A (Y/N) | Binomial |
| Updated immunization schedule (Y/N) | Binomial |
| Catch-up vaccination provided (Y/N) | Binomial |
| Uptake of contraception among eligible women (Y/N) | Binomial |
| Type of contraception provided | Categorical |

### Variables collected 5 weeks after the campaign

| Variable | Type of variable |
| --- | --- |
| Enrolled into chronic care for HIV (Y/N) | Binomial |
| Enrolled on ART (Y/N) | Binomial |
| Brought sputum to facility within ≤ 5 days (Y/N) | Binomial |
| Result of sputum examination (Pos/Neg) | Binomial |
| Absolute number tested HIV-positive during this period from the 5 villages with campaigns and from 5 control villages (without campaign) | Count |

### Collection and processing of data

During the testing campaign, data will be collected by the nurse, using a paper-based collection form (see annex 2). After the campaign a list of all clients tested HIV-positive and/or with a positive TB-screening will be created. Based on this list, data managers will visit the health centers 5 weeks after the campaigns and check in the registers, if these clients came to the facility to be enrolled into chronic HIV/AIDS care and/or to bring their sputum for smear-examination. (As mentioned in annex 1, patients who are expected to bring sputum to the facility and who do not appear at the facility within 5 days have to be traced immediately – bringing sputum after 5 days (through tracing or not) is considered as a negative outcome in the analysis).

Based on information of HTC- and pre-ART/ART-registers, data managers will collect data on absolute numbers newly tested HIV-positive and newly enrolled in chronic HIV/AIDS care during the 4 weeks after the campaigns from each cluster.

Data will subsequently be entered into an EpiData 3.1® database, using double-entry in order to ensure accuracy of data. On the paper records the patient-names appear (as they are needed to verify if the patient came for further care at the facility). However, the names are replaced by a code-identifier before being entered for analysis. The electronic database does not contain any patient-names. The paper-records are stored in locked cupboards at the two SolidarMed site-offices.

## Statistical Analysis

All primary outcomes are binary variables. Absolute counts and proportions will be reported. To assess differences in primary outcomes, adjusted logistic regression will be used. All logistic regression models will be adjusted for clustering using the cl-command in STATA.

## Selection biases and confounders

### Cluster (Health Center) Level

The randomization of the clusters is particularly prone to biases due to the very low number. The main bias is that some catchment areas may have better access to services, may be more accessible for HTC or may have a different HIV-prevalence. In order to minimize this bias, health centers were matched based on their routine performance in terms of numbers accessing HTC per month, numbers testing positive per month and numbers enrolled into chronic HIV/AIDS care per month.

### Subcluster (village) level

Some campaigns may be conducted in communities that are particularly easy to access or that are particularly open to HTC-services. Other communities may have had a HTC-campaign just a few months ago. Some villages may be chosen because they are particularly suitable for one kind of intervention (door-to-door or pitso). In order to minimize these selection biases, the following procedure has been chosen: Before having been introduced of the kind of campaign (door-to-door or pitso) that will be conducted, the health center team provides a list of 10 villages that fulfill the eligibility criteria. From this list only 5 are randomly chosen (see subsection on selection of villages) for the campaigns.

### Individual level

On an individual level, the traditional way of HTC-campaigns (pitso) is a very biased approach as someone has to show up at the pitso in order to get the services. Door-to-door will probably reach other people as compared to pitso. However, it is part of the study-hypothesis that a different population will be reached using door to door. Differences in age and gender for example will be assessed as secondary outcomes. As the pitso is usually held in the center of the village (chief’s place), the door-to-door campaigns also start at the center and move towards the periphery.

## Study-flow

5 villages randomly chosen out of the 10 selected villages

5 villages randomly chosen out of the 10 selected villages

Campaigns are held according to the schedule.

# Ethical Considerations:

Selection of 10 villages per health center

6 Health Centers pitso

6 Health Centers door

Remaining outcomes are collected 5 weeks after campaigns were held

5 villages randomly chosen out of the 10 selected villages

## Facilities and teams conducting testing campaigns

All facilities involved are certified ART-centers where primary health care services, HTC and ART are provided according to the standards of the Ministry of Health and Social Welfare of Lesotho. The facilities work according to good practice of keeping patient’s intimacy and confidentiality. The teams are constituted of experienced lay-counselors and ART-nurses who practice HTC according to national standards.

## Informed consent

All patients will be approached for informed consent to use their data for analysis (see informed consent form annex 3). Only patients providing informed consent will be enrolled in the study. Patients refusing consent will benefit from the same services and quality of care as patients who agree in participation. In the case of children < 16 years, informed consent has to be provided by an adult care-taker. Persons may withdraw from the study at any moment without any diadvantages.

## Compensation

This study causes no any additional risk or cost to the patients. Therefore we do not pay compensation to the participants.

## Confidentiality

Confidentiality and privacy are ensured. The paper-records contain patient-names and are stored in a locked cupboard of the SolidarMed offices. The database containing the study variables is on a password-protected computer and the files are only accessible to the investigators and data managers. The database used for analysis will be anonymous, codes as unique identifier.

# Annex 1: Standards of procedures

**Standard Operation Procedures for HTC-campaigns**

- fill-in a file for each client approached for the provision of services
- follow the order of services provided as outlined below

| **Number** | Consecutive numbering. Each team starts every day at 1 |
| --- | --- |
| **Name** | Write Full Name |
| **Resident in CA?** | If the client is resident in the catchment area of the health center where the campaign takes place 🡪 tick Yes |
| **Blood Pressure** | Provide this service to all patients >18 years and write measured blood-pressure into the case. For children 🡪 leave empty.  If blood-pressure ≥140/90 mmHg 🡪 refer to health center for follow-up |
| **HTC accepted** | Propose HTC to all clients (independent of age) who are not already confirmed HIV-positive. All clients agreeing to HTC must fill out the national HTC-consent form!   - if client accepts HTC 🡪 tick “Yes” - if client does not accept HTC 🡪 tick “No” - if client states to be HIV-positive 🡪 tick “ICDP” - if it is a child < 12 years without her/his care-taker 🡪 tick “no guard” - if client is currently in the window-period (<3months since last HIV-test, as confirmed in bukana) 🡪 tick “window”   In case of children < 18 months with a positive HIV-antibody test, the care-taker must be counselled to seek care at the health center for a PCR-confirmation test. |
| **1^st^ time tester** | - if client states that he has never tested before 🡪 tick “Yes” - if client states that he once tested before 🡪 tick “No” - if client is not tested for any reason 🡪 tick “NA” |
| **Test result** | Copy result from test: Positive or Negative or Indeterminate  If the test is positive, the national HTC referral form must be filled out. A list of all newly patients tested positive must be provided to the health center. Patients who do not appear at the health center within 1 month must be traced by the health center (using the SolidarMed tracing fund) |
| **WHO-stage** | Stage all clients with a positive HIV-test according to WHO-staging (see annex) |
| **CD4-count** | Perform CD4-count (PIMA) to all clients who test HIV-positive and note the count in the field. |
| **Height/Weight** | Put height in cm and weight in kg |
| **Blood Glucose** | Provide blood-glucose testing to all patients > 18 years and write measured blood glucose into the case. For children 🡪 leave empty   - If blood-glucose ≥7mmol/l 🡪 refer to health center for follow-up - If blood-glucose ≥ 17mmol/l 🡪 transport to hospital immediately |
| **Deworming** | **Eligible for deworming:**  age 1-5 years and no documented deworming for the last 6 months   - If eligible and deworming supplied 🡪 tick Yes - If eligible and deworming refused by client 🡪 tick No - If not eligible 🡪 tick N/A |
| **Immunization** | **Eligible clients:**  Children or women of child-bearing age who are not vaccinated according to the national immunization-schedule   - If eligible and catch-up vaccination supplied 🡪 tick Yes - If eligible and catch-up vaccination refused by client 🡪 tick No - If not eligible 🡪 tick N/A |
| **Vitamin A** | **Eligible clients:**  Children or women of child-bearing age who have not received Vitamin A according to national schedule   - If eligible for Vitamin a and Vitamin A supplied 🡪 tick Yes - If eligible for Vitamin A but refused to take Vitamin A 🡪 tick No - If not eligible 🡪 tick N/A |
| **Uptake FP** | **Eligible clients:**  All women of child-bearing age.   - If eligible and accepted counselling for one FP-method (Pill, injection, coil) 🡪 refer to health center and tick Yes - If eligible but does not accept counselling for FP 🡪 tick No - If not eligible 🡪 tick N/A   All clients should receive condoms! However, for the study, distribution of condoms is not recorded as FP. If only condoms are given, but the women does not accept counselling for one of the following methods: Pill, injection or coil, then tick No. |

| **TB-screening** | TB-screening has to be done to all clients (HIV-negative and HIV-positive).  **Do screening as follows:**  **HIV-negative adult/adolescent person:**   - Are you coughing since more than 2 weeks? - Have you lost weight? - Do you have drenching/soaking sweats at night? - Do you have fevers?   🡪 if the answer of any of these questions is yes 🡪 tick TB-screening positive, assess the patient and provide 3 sputum bottles.  **HIV-negative infant/child:**   - Is the child coughing since more than 2 weeks? - Has the child unexplained fever for more than 3 days? - Does the child have failure to thrive/faltering growth or signs of severe malnutrition? - Has the child been in contact with someone with smear + TB?   🡪 if the answer of any of these questions is yes, tick screening positive, assess child for any danger signs and refer for MD-assessment.  **HIV-positive adult:**  Are you coughing?   - Have you lost weight? - Do you have drenching/soaking sweats at night? - Do you have fevers?   🡪 if the answer of any of these questions is yes, tick TB-screening positive, assess the patient and provide 3 sputum bottles.  **HIV-positive infant/child:**   - Is the child coughing? - Has the child fever? - Does the child have failure to thrive/faltering growth or signs of severe malnutrition? - Has the child been in contact with someone with smear + TB?   🡪 if the answer of any of these questions is yes, tick screening positive, assess child for any danger signs and refer for assessment by medical doctor at the hospital.  **Sputum-collection:**   1. Instruct TB-suspect on sputum collection 2. Fill out sputum request form 3. TB-suspect shall provide one on-the-spot sputum outside the house 4. TB-suspect shall bring the 3 bottles to the HC the following day 5. Note the clients in the TB-suspect register at the health center 6. Instruct health center to trace the TB-suspect, if he/she does not bring the sputum bottles within 5 days (the health centers can use the SolidarMed tracing fund) |
| --- | --- |
| **Sputum bottles given** | Provide 3 sputum bottles to each adult patient with positive TB-screening. If sputum-bottles provided 🡪 tick Yes. |
| **Informed consent** | All clients (no matter if they agree to HTC or not) have to fill out an informed consent.   - if they agree to provide their anonymous data for the study they shall tick “kea lumela/tekena” and either sign or provide finger-print - if they disagree to provide their anonymous data for the study they shall tick “Kea latola/tekena” |

# Annex 2: Study-questionnaire (data-collection tool)

| **Number** | **Name** | **Resident in CA?** | | **Sex** | | **Age** | **BP** |
| --- | --- | --- | --- | --- | --- | --- | --- |
|  |  | ⬜ Y | ⬜ N | ⬜ F | ⬜ M |  | mm/Hg |

| **HTC accepted** | | | | | | | **1st time tester** | | | | **Test Result** | | | |
| --- | --- | --- | --- | --- | --- | --- | --- | --- | --- | --- | --- | --- | --- | --- |
| ⬜Y | ⬜ N | | ⬜ ICDP | ⬜ no guard | | ⬜ window | ⬜ Y | ⬜ N | | ⬜ N/A | ⬜ Pos | ⬜ Neg | | ⬜ Ind |
|  | |  | | |  |  |  | |  | |  |  | |  |
| **WHO-stage** | | | | | **CD4** | | **TB-screening** | | | | **Sputum bottles given** | | | |
|  | | | | | cells | | ⬜ pos | | ⬜ neg | | ⬜ N | | ⬜ Y | |

| **Height** | | | | **Weight** | | | | **Blood-glucose** | | | | |
| --- | --- | --- | --- | --- | --- | --- | --- | --- | --- | --- | --- | --- |
| cm | | | | kg | | | | mmHg | | | | |
|  | | |  | | |  | | | |  | | |
| **Deworming** | | | **Immunization** | | | **Vitamin A** | | | | **Take-up FP** | | |
| ⬜ Y | ⬜ N | ⬜ N/A | ⬜ Y | ⬜ N | ⬜ N/A | ⬜ Y | ⬜ N | | ⬜ N/A | ⬜ Y | ⬜ N | ⬜ N/A |

**Outcomes (leave empty, except if already registered icdp)**

| **Brought sputum** | | **Sputum-result** | | **Enrolled on pre-ART** | | **Enrolled on ART** | | **Pre-ART** | **ART-Nb** |
| --- | --- | --- | --- | --- | --- | --- | --- | --- | --- |
| ⬜ Y | ⬜ N | ⬜ Neg | ⬜ Pos | ⬜ Y | ⬜ N | ⬜ Y | ⬜ N |  |  |

# Annex 3: Informed Consent form

Tumello ho tsoa ho mosebeletsuoa

Ntate/’M’e ea khabane

U lumetse ho hlahlobela kokoana - hloko ea HIV, ka hoo re u atamela ho kopa hore litaba tsa hao tsa lilemo le botona/bots’ehali le sephetho sa hau le litaba tsa ts’alo morao setsing li ka sebelisoa bakeng sa lipatlisiso. Sepheo sa boithuto ke ho tla fumana na ke mokhoa o fe oa thlahlobo o ka sebetsang hantle mona Lesotho. Ha u lumela litaba tsa hao litla sebelisoa ntle le ho hlalosa boleng esita le mabitso a hau lipatlisisong tsa rona. Empa ha u latola seo ha sena ho etsahala. U lokolohile ho hana kapa ho lumela. U lumetse kapa u latotse u ntse u tla fumana lits’ebeletso tsa rona tse hloahloa.

Motse le letsatsi ___________________________________

Kea lumela/tekena ________________________________

Kea latola/tekena __________________________________

*English Translation:*

*Agreement from the client*

*Mister or Madam*

*Today you have agreed to accept the services our team provides in your village. We therefore ask you to allow us to use the information you provided to us in the questionnaire for research purposes. The main aim of the research is to find out the effectiveness of different kinds of HTC-campaigns in Lesotho. If you agree to participate, we would therefore use your data anonymously (without stating who you are). If you disagree we will not use your data for analysis. You are free to agree or disagree. No matter if you agree or disagree, we will undertake all efforts to provide you the best services possible.*

*Village/Date _________________________*

*I agree _____________________________*

*I disagree ___________________________*

1. Names of villages are put in a bag and then a blinded person draws the five that will conduct the campaigns. The other five automatically fall into the control-group. [↑](#footnote-ref-1)
2. Hayes RJ, Bennett S: **Simple sample size calculation for cluster-randomized trials.** *Int J Epidemiol* 1999, **28**:319-326 [↑](#footnote-ref-2)
